# Acupuncture for thyroid nodule treatment: A protocol of systematic review and meta-analysis of randomized clinical trials

**DOI:** 10.1097/MD.0000000000022276

**Published:** 2020-10-02

**Authors:** Qi Chen, Jun Zhou, Xinxia Zhang, Lizhen Wang, Botong Yang, Jia Xia, Min Zhong, Xiaoming Tang

**Affiliations:** a Hospital of Chengdu University of Traditional Chinese Medicine; b Chengdu University of Traditional Chinese Medicine, Chengdu, Sichuan, China.

**Keywords:** acupuncture, meta-analysis and systematic review, protocol, thyroid nodules

## Abstract

**Introduction:**

Thyroid nodules are scattered lesions caused by abnormal local growth of thyroid cells. In recent years, their prevalence rate has been rising gradually, and the probability of cancerations has also been increasing gradually. Therefore, we must pay more attention to them and carry out early intervention. However, at present, most of the intervention measures for patients with thyroid nodules are mainly clinical observation and follow-up, and no clear and effective drug intervention therapy has been proposed. The curative effect of acupuncture on thyroid nodules has been proved clinically. However, as there is no clear mechanism of action, no specific operation methods or Suggestions, it is necessary to make a systematic evaluation of acupuncture therapy, so as to lay a foundation for further research in the future.

**Methods and analysis:**

The following databases will be searched from their inception to June 2020: Electronic database includes PubMed, Embase, Cochrane Library, Web of Science, Nature, Science online, Chinese Biomedical Database WanFang, VIP medicine information, and China National Knowledge Infrastructure (CNKI). Primary outcomes: Color ultrasound of thyroid and cervical lymph nodes, FT3, FT4, TSH, TGAB, TPOAB, insulin resistance index (HOMA-IR). Data will be extracted by 2 researchers independently, risk of bias of the meta-analysis will be evaluated based on the Cochrane Handbook for Systematic Reviews of Interventions. All data analysis will be conducted by data statistics software Review Manager V.5.3. and Stata V.12.0.

**Results:**

The results of this study will systematically evaluate the efficacy and safety of acupuncture therapy for patients with thyroid nodule.

**Conclusion:**

Through the systematic review of this study, the evidence of the treatment of thyroid nodule by acupuncture has been summarized so far, so as to provide guidance for further promoting the application of acupuncture therapy in patients with thyroid nodule.

**Ethics and dissemination:**

This study is a systematic review, the outcomes are based on the published evidence, so examination and agreement by the ethics committee are not required in this study. We intend to publish the study results in a journal or conference presentations.

**Open Science Fra network (OSF) registration number:**

August 18, 2020. osf.io/uzck4. (https://osf.io/uzck4)

## 1 Introduction

Thyroid nodule is a common disease in endocrinology department, and its prevalence rate has increased greatly in recent years. Epidemiological studies have shown that in areas with adequate iodine, the prevalence of palpable thyroid nodules is about 5% in women and 1% in men. High-resolution ultrasound can detect thyroid nodules in 19% to 68% of the randomly selected population.^[1]^

Clinical handling of thyroid nodule can be roughly divided into 2 methods of surgical and nonsurgical, most benign thyroid nodules and no obvious clinical symptoms can adopt the method of observation of follow-up, to have cancer diagnosis, clinical symptoms of thyroid nodule surgical treatment,^[2]^ and out of the neck discomfort, independent functions, beautiful problems or intend to factors such as patients, part of benign thyroid nodules feasible thyroid hormone suppression therapy and percutaneous ethanol injection treatment, thermal ablation and radioactive iodine treatment.^[3]^ At present, the clinical application of traditional Chinese medicine (TCM) in the treatment of benign thyroid nodules mainly includes 2 categories: internal treatment (oral administration of TCM) and external treatment (external application of TCM, acupuncture, auricular injection, acupoint injection, TCM ion introduction, etc).^[4]^ Among them, TCM internal treatment for benign thyroid nodules has a solid theoretical foundation, widely used, good curative effect, but there are some shortcomings, such as long drug cycle, tedious cooking steps, high treatment cost and so on. However, the application of TCM external therapy in the treatment of thyroid nodules is relatively rare, but its effectiveness and safety have been proven clinically, but lack of exact theoretical basis. Therefore, this study intends to use the systematic evaluation and meta-analysis of acupuncture therapy for thyroid nodule treatment, to evaluate its efficacy and safety, and to explore its possible theoretical basis.

## 2 Methods

### 2.1 Study registration

The protocol has been registered in OSF (Open Science Framework) Preregistration. August 18, 2020.osf.io/uzck4 (https://osf.io/uzck4). The protocol will follow the statement guidelines of Preferred Reporting Items for Systematic Reviews and Meta-Analyses Protocols (PRISMAP),^[5]^ Changes will be reported in the full review as required.

### 2.2 Inclusion and exclusion criteria for study selection

#### 2.2.1 Inclusion criteria

The inclusion criteria were all randomized controlled trials (RCTs), with acupuncture as the main treatment for thyroid nodules. The language of the trials to be included only Chinese or English.

#### 2.2.2 Exclusion criteria

Following studies will be excluded:

1. Cancer (thyroid cancer and metastatic thyroid cancer), malignant transformation of thyroid nodules 50% to 90% risk, thyroiditis;
2. Thyroid nodules ≥4 cm;
3. If the treatment of other diseases requires long-term adherence to certain drugs, which may affect thyroid function;
4. A history of neck surgery and radiation therapy;
5. Pregnant or lactating women;
6. Patients with serious primary diseases, such as hematopoietic system, cardio-cerebrovascular system, liver, kidney, and mental system;
7. Susceptible to allergies;
8. Non - RCTs and Quasi - RCTs
9. Case Series and Reviews
10. Animal research.

### 2.3 Types of participants

The subjects included patients diagnosed with thyroid nodules regardless of their size (≦4 cm) or abnormal thyroid function. All patients should be treated with acupuncture, either primarily or in combination with other conventional therapies. There is no sense of gender, race, or education.

### 2.4 Experimental interventions

Acupuncture should be the main treatment. Acupuncture points: main points: Fenglong, sea of blood, Tai Chong, he Gu Xie method, shan, Dazhui, People ying, Wan, Quchi ping Ping Ping xie.

### 2.5 Control interventions

Intervention measures include: follow-up observation, nondrug intervention (such as diet, exercise, smoking cessation, abstinence from alcohol, reasonable schedule, etc), acupuncture therapy. Joint interventions are allowed as long as all groups in a randomized trial receive the same joint intervention.

### 2.6 Types of outcome measures

#### 2.6.1 Main outcomes

1. Thyroid color doppler ultrasound;
2. Color doppler ultrasound of cervical lymph nodes;
3. Thyroid function (FT3, FT4, TSH, TGAB, TPOAB).

#### 2.6.2 Additional outcomes

Insulin resistance index (HOMA-IR).

## 3 Data sources

### 3.1 Electronic searches

The following data bases will be searched to identify eligible studies: PubMed, Embase, Cochrane Library, Web of Science, Nature, Science on line, Chinese Biomedical Database WanFang, VIP medicine information, and China National Knowledge Infrastructure (CNKI). The time range is: the starting time is determined according to the first literature available, and the deadline is August2020.

### 3.2 Other search resources

**Figure 1:**
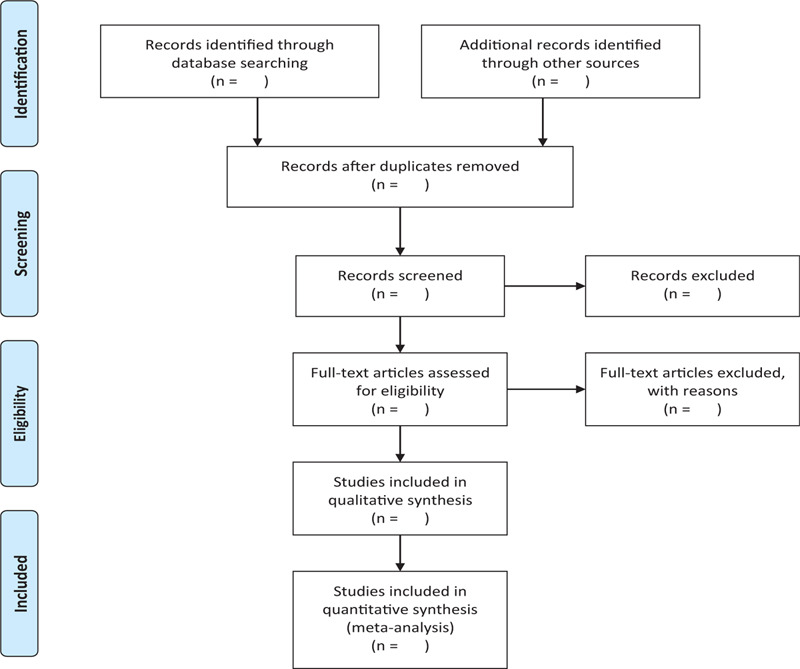
The research flowchart. This figure shows the Identification, Screening, Eligibility and Included when we searching articles.

In order to get more complete evidence, we will also retrieve other related documents by manually, such as medical textbooks, clinical laboratory manuals and so on. If it is necessary, we will contact with trail author to obtain the latest clinical data. Moreover, studies associated with the review will be identified via evaluating related conference proceedings. The research flowchart is shown in Figure 1.

### 3.3 Search strategy

The following search terms will be used: randomized controlled trial/RCT; thyroid nodules/Affection of thyroid nodule; traditional Chinese medicine/TCM; acupuncture/acupuncture therapy/acupuncture treatment. different retrieval strategies in Chinese and foreign databases will be used. Language restrictions are Chinese and English. There is no publication restriction. Here we take the search strategy in PubMed as an example and list in Table 1.

**Table 1:**
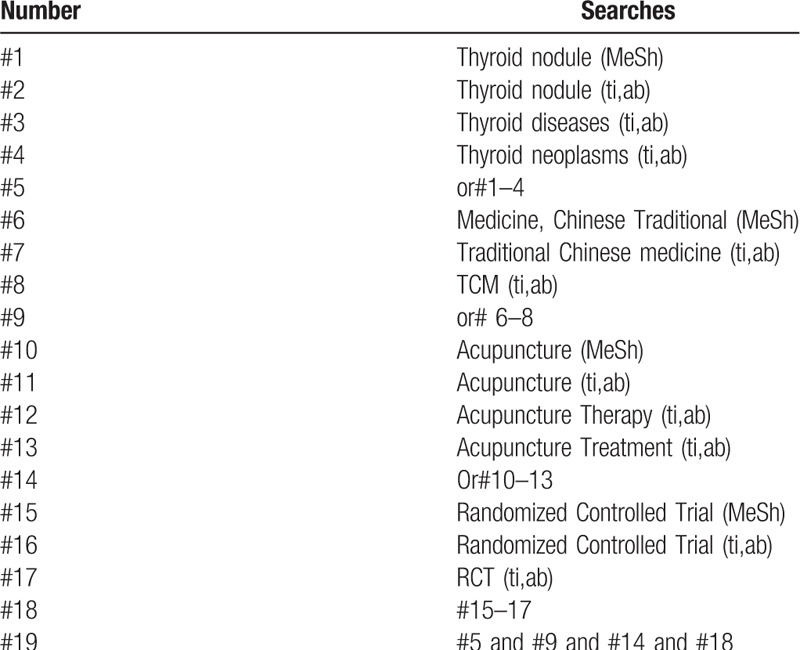
Search strategy sample of PubMed.

## 4 Data collection and analysis

### 4.1 Study selection

All articles in the search results were independently evaluated by 2 independent researchers (QC, JZ) according to inclusion and exclusion criteria. Reviewers will then independently extract and collect the data included in the study using predesigned data collection forms. Discrepancies will be discussed and resolved by consensus with a third author (XX).

### 4.2 Data extraction and management

The following information will be extracted from each study:

(1) Normal test characteristics: title, author, year.
(2) baseline data: sample size, age, gender, diagnostic criteria, course of disease.
(3) interventions: acupuncture therapy, control of intervention details, intervention.

If the information is not enough, we will contact experts and authors in this field to get relevant information.

### 4.3 Assessment of the reporting quality and risk of bias

The risk of bias will be assessed by 2 independent authors (QC and JZ), together with completing the STRICTA checklist.^[6]^ The Cochrane System Evaluator's Manual give the evaluation criteria for authors to evaluated the RCTs’ quality. Assessing the risk of bias:

1. random sequence generation;
2. allocation concealment;
3. blinding of participants and personnel;
4. blinding of outcome assessment;
5. incomplete outcome data;
6. selective outcome reporting;
7. other bias.

Any disagreement will be discussed or consulted with a third reviewer. Each them will be described from 3 levels: “high risk,” “low risk” and “unclear.”

### 4.4 Measures of a treatment effect

The dichotomous outcomes will be expressed by the Odds ratio, while the continuous data will use the Standardized mean difference. All these outcomes report 95% confidence interval.

### 4.5 Management of missing data

We will take the method of contacting corresponding authors to obtain the missing data. If there is no response, we will analyze only the available data and describe the reason and impact of this exclusion in the paper.

### 4.6 Assessment of a reporting bias

The bias of publication will be explored through funnel plot analysis. If the funnel plot show asymmetry, it will be evaluated via the Egger and Beg tests, and *P* value <.05 means the publication bias is significant.

### 4.7 Assessment of heterogeneity

There are 2 main methods for testing heterogeneity, namely graphical method (funnel plot, forest plot) and statistical test (*Q* value statistic test, *I* ^2^ statistic test, *H* statistic test). The *I* ^2^ statistic test method shows us When *I* ^2^ is 0, it means that studies are completely homogeneous, If *I* ^2^ > 50%, it indicates there is heterogeneity in studies.

### 4.8 Data synthesis and grading of quality of evidence

The results of the study will be analyzed by RevMan 5.0 software provided by Cochrane collaborate on network. The binary data will be expressed by the odds ratio, while the continuous data will use the mean difference. To test the heterogeneity of the research results, when the *I* ^2^ < 50% or *P* > .1, the heterogeneity is significant. The random effect model was used for the meta-analysis, otherwise, we choose the fixed effect model.

### 4.9 Subgroup analysis

 

### 4.10 Sensitivity analysis

Sensitivity analysis can not only assess the stability and reliability of the conclusions of the Meta-analysis, but also assess whether the changes in the results are related to the impact of a single study. If the stability of the conclusion is poor, we can achieve when the heterogeneity test results are heterogeneous, we need to clarify the source of the heterogeneity by subgroup analysis. The effects of different types of therapy including design scheme, severity of illness, age, sex, and mild or severe T2DM were analyzed. We will also delete low-quality and/or medium-quality studies to check the robustness of the results.

The purpose of increasing stability by changing the analysis model, inclusion and exclusion criteria, or excluding a certain type of literature.

### 4.11 Ethics and dissemination

We will publish the system review results in peer-reviewed journals, disseminated in meetings or in peer-reviewed publications. Aggregated published data will be used to exclude data of individuals, so there is no need for obtaining the ethical approval or patients’ informed consent.

## 5 Discussion

The cause of thyroid nodules has not been clearly defined yet, but it can be summarized as follows:

(1) Pathogenic factor:
  - The incidence of thyroid nodules in women is significantly higher than that in men.
  - The incidence of thyroid nodules increases with age.
  - Thyroid nodules and thyroid cancer are associated with mutations, activation, inhibition, and deletion of oncogenes and tumor suppressor genes.
  - The higher the urine iodine value, the higher the detection rate of ultrasonic thyroid nodules.
  - People exposed to radiation are at risk of developing differentiated thyroid cancer over the next 40 years or even throughout their lives.
  - Smoking and drinking alcohol can increase the incidence of thyroid nodules.
(2) Risk factors:
  - In addition to iodine nutrition, many factors, including lifestyle and environmental pollution, can also affect the incidence of thyroid nodules.
  - Elevated estrogen levels may increase the risk of thyroid nodules.
  - Insufficient sleep may affect the generation and growth of thyroid nodules;
  - Insulin has an obvious promoting effect on angiogenesis and may stimulate tumor growth by increasing blood supply.[^7^,^8^]

Acupuncture as a treatment the curative effect of treatment of benign thyroid nodules is has a certain theoretical basis, research by AG7A8 type infrared thermal image instrument to explore the mechanism of acupuncture treatment of thyroid nodule, it is concluded that acupuncture can increase the tumors had local blood circulation, promote metabolism, thus to nodules, tumors and tumors had softened, shrink or even disappear.^[9]^ Some researchers also believe that the local acupoint river of the superficial pricking mass can make the skin chemoreceptor receive the stimulation of acupuncture and regulate the cerebral cortex function to form a benign excitatory focus. By activating macrophages in the body to play their phagocytosis function, pathological excitatory foci are inhibited, which promotes the softening, atrophy and disappearance of tumors, masses and nodules.^[10]^ In addition, acupuncture can improve the body's neuroendocrine system and immune system to stimulate the innate immune ability of the body, thus normalizing abnormal indicators and pathological changes.^[11]^

In recent years, clinical experience has proved that acupuncture is an effective and safe therapy for thyroid nodules, and relevant studies have proposed its possible mechanism of action, but its mechanism is not clear, most of the literature has not clearly pointed out the research mechanism of acupuncture for benign thyroid nodules. Therefore, it is very necessary to carry out a standardized and unified experimental design to evaluate the efficacy in clinical studies, which can not only guide the application of acupuncture therapy in thyroid diseases, but also explore the mechanism of acupuncture therapy in the treatment of thyroid nodules.

To sum up, systematic review and meta-analysis are helpful to determine the standard treatment of thyroid nodule with acupuncture and reduce the waste of medical resources, patients’ time and money. This study can not only provide the basis for the release of thyroid nodule treatment guidelines, but also promote the application of acupuncture therapy, so as to benefit more patients.

## Author contributions

**Conceptualization:** Qi Chen, Jun Zhou, Xinxia Zhang, Lizhen Wang, Botong Yang, Jia Xia, Min Zhong, Xiaoming Tang

**Data curation:** Botong Yang, Jia Xia.

**Formal analysis:** Qi Chen, Jun Zhou, Lizhen Wang.

**Methodology:** Qi Chen, Jun Zhou, Xinxia Zhang;

**Project administration:** Xinxia Zhang.

**Resources:** Botong Yang, Qi Chen, Jun Zhou, Min Zhong, Xiaoming Tang.

**Software:** Lizhen Wang, Qi Chen, Jun Zhou, Min Zhong, Xiaoming Tang.

**Supervision:** Xinxia Zhang.

**Writing – original draft:** Qi Chen.

**Writing – review & editing:** Min Zhong, Xinxia Zhang.
